# Lupus mastitis - peculiar radiological and pathological features

**DOI:** 10.4103/0971-3026.50834

**Published:** 2009-05

**Authors:** Abdul Majid Wani, Waleed Mohd Hussain, Mohamed I Fatani, Bothaina Abdul Shakour

**Affiliations:** Department of Radiology, Hera General Hospital, Makkah-10513, Saudi Arabia

**Keywords:** Biopsy, lupus mastitis, lupus profundus, mammography, fine needle aspiration cytology, systemic lupus erythematosus

## Abstract

Lupus mastitis is a form of lupus profundus that is seen in patients with systemic lupus erythematosus. It usually presents as a swelling (or swellings) in the breasts, with or without pain. The condition is recurrent and progresses along with the underlying disease, with fat necrosis, calcification, fibrosis, scarring, and breast atrophy. Lupus mastitis is often confused with malignancy and lymphoma and, in our part of the world, with tuberculosis. Confusion is especially likely when it occurs in an unusual clinical setting. In this article, we present a case that presented with unique radiological, pathological, and clinical features. Awareness of the various manifestations of lupus mastitis is essential if unnecessary interventions such as biopsies and surgeries, and their consequences, are to be avoided.

## Introduction

Systemic lupus erythematosus (SLE) is a multisystem, autoimmune disorder. Involvement of the subcutaneous fat was termed as lupus profundus by Iregang.[1] Lupus mastitits is the term for breast involvement; this condition occurs only rarely[2,3] and is often confused with malignancy, lymphoma, and tuberculosis. The typical presentation is with recurrent painful swellings that respond to antimalarials like chloroquine and hydroxychloroquine. We present an interesting case of lupus mastitis that had unusual radiological and pathological features.

## Case Report

**Figure 1:**
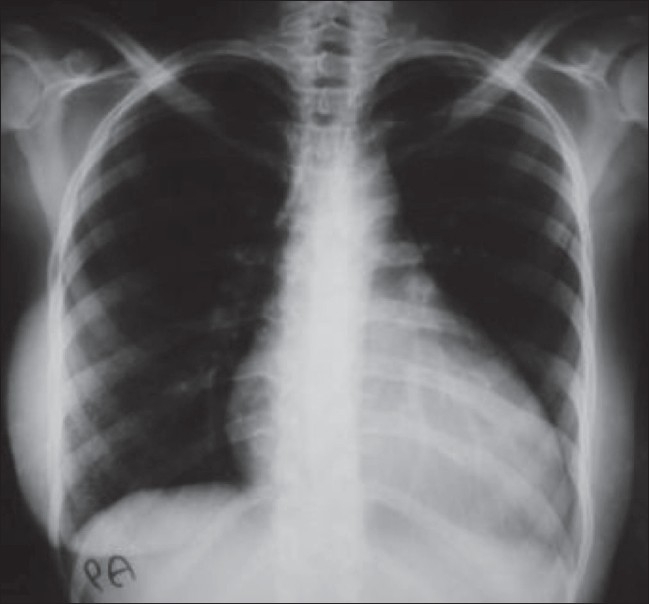
X-ray chest shows diffuse calcifications in both breasts

**Figure 2:**
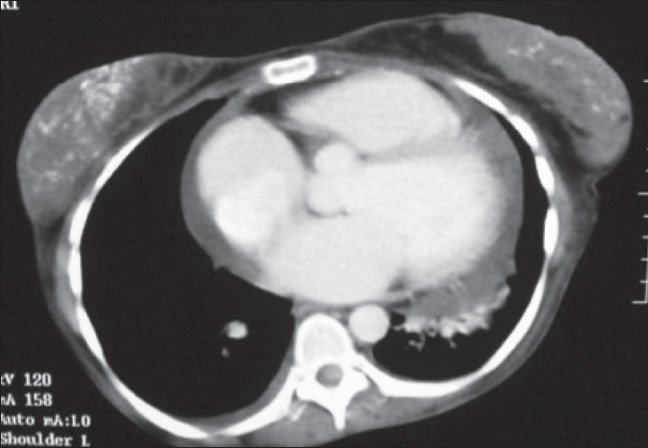
CT scan of the chest shows calcifications in both breasts and minimal pericardial effusion

A 24-year-old woman was admitted with complaints of fever, weight loss, decreased appetite, swelling in the left submandibular region, and painful swelling of the left breast. She was a known case of SLE who was on regular follow-up with the rheumatology department for the last 13 years and had her disease under control with prednisone, hydroxychloroquine, and azathioprine. On examination she was emaciated, with a body mass index of 16. She had mild pallor. There was an indurated swelling, consistent with matted lymph nodes, in the left submandibular region. The scar of a previous biopsy (performed 6 years back at another hospital) was present and the patient reported that she had received antituberculous medication for 1 month at that time. Two 1 × 1.5 cm lymph nodes were present in the left axillary region. The left breast revealed multiple lumps, the biggest being 4 × 3 cm in size. Cardiac examination revealed muffled heart sounds. A palpable liver was detected on abdominal examination. Complete blood count showed normochromic normocytic anemia and leucopenia. Serum chemistry was normal, except for a low albumin level of 2.6 gm/dl and mild elevation of transaminases. The ESR was 115 mm and the serum ferritin level was > 2000 μg/dl. A septic screen was negative, as was the Mantoux test. A plain radiograph of the chest showed cardiomegaly and diffuse calcifications in the region of the breast [Figure 1]. Echocardiography revealed mild pericardial effusion and an ejection fraction of 20%. CT scans of the chest and abdomen were performed and showed mild pericardial effusion and unusual calcifications in the breasts [Figure 2]. CT scan also ruled out any abdominal or mediastinal lymphadenopathy. A biopsy of the submandibular swelling showed features consistent with panniculitis.

**Figure 3:**
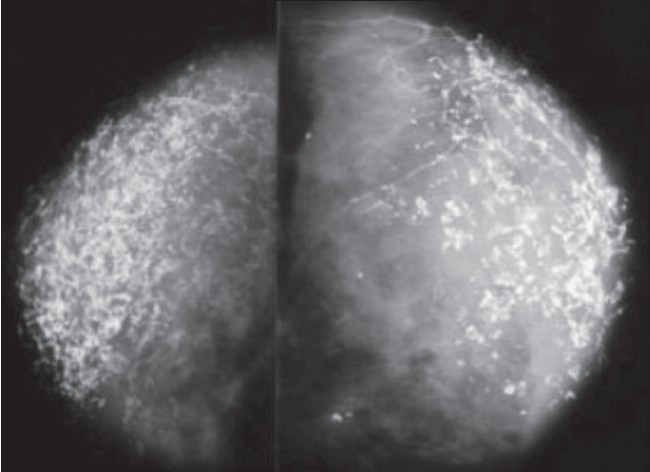
Mammography shows unusual and diagnostic calcifications

**Figure 4:**
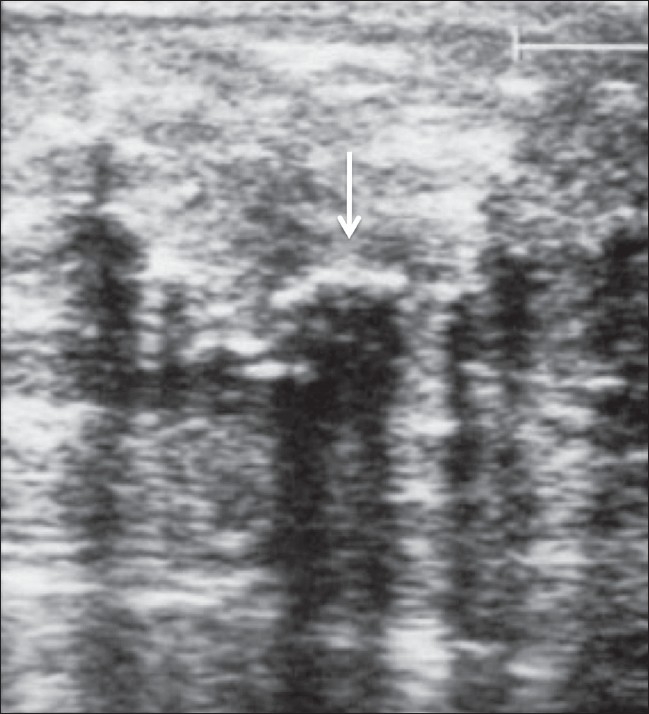
USG of the breast shows diffuse calcifications with acoustic shadowing (arrows)

In view of the constitutional symptoms, marked emaciation, past history of inadequate treatment for tuberculous lymphadenitis, and the breast swelling, we considered the possibilities of tuberculosis, lymphoma, and breast malignancy. Mammography showed unusual findings [Figure 3]. Breast USG showed diffuse calcifications [Figure 4]. A core biopsy of the breast was performed, which revealed panniculitis. Due to the high prevalence of tuberculosis in our part of the world and the clinical features of the patient, antituberculous medication was prescribed and the patient was discharged. To our surprise, she improved; she gained weight, became ambulatory, the swelling in the neck decreased in size, and her constitutional symptoms subsided. However, 3 months later, the patient again presented with pain and swelling of her right breast and was referred to the surgical department, where physical examination revealed multiple lumps of varying sizes in both breasts, without any nipple discharge or involvement of the overlying skin. Fine-needle aspiration (FNA) of one of the swellings was performed and showed degenerated fat cells, lymphocytes, and foci of calcification. The pain subsided within a few days.

The patient is being followed up; the breast swellings are nodular at present and she is doing well. We did not repeat biopsy as trauma is reported to precipitate and worsen lupus mastitis.

## Discussion

SLE is an autoimmune disease in which pathogenic autoantibodies are the primary cause of tissue damage. People of all ages, all ethnic groups, and both sexes are affected, but 90% of the cases occur in women of childbearing age.[4] Kaposi first proposed the term ‘lupus panniculitis’ in 1883.[5] Irgang introduced the term ‘lupus profundus’ for the inflammation of the subcutaneous fat that occurs in 2–3% of SLE patients.[1,6] Involvement of the breasts, called lupus mastitis, is rare. According to some sources, not more than 10 cases (and according to Niger *et al*., not more than 16 cases) have been reported to date.[2,3] The disease, as expected, is more common in women in the age-group of 20–50 years, though cases have also been reported in men.[7,8] Patients may present with recurrent breast swellings, with or without pain. Clinically, multiple subcutaneous nodular swellings are palpable, with or without involvement of the overlying skin.[9] The pathophysiology of lupus mastitis is unknown. One belief is that local trauma, including that due to biopsy, may precipitate lupus mastitis and that it may herald SLE.[10] However, in our opinion (and as also reported by others) lupus mastitis is an extension of the inflammatory process that involves the overlying skin. If the breasts are involved in the absence of skin involvement, the process may be the result of vasculitis.[2] Traumatic procedures like biopsy may worsen[3] the condition and it is advisable to avoid biopsy if the diagnosis can be established with the clinical and radiological features (especially the unusual mammographic calcification) as was possible in our patient. FNA will be helpful if there is a doubt about the diagnosis or when the swelling is localized. Degenerated fat cells with scattered foci of calcification and lymphocytic predominance are the classic findings seen on microscopic examination. Cytology has been reported to be capable of differentiating between granulomatous mastitis and other benign lesions or malignancies.[11]

Histological findings in lupus panniculitis include mainly: 1) hyaline fat necrosis, 2) lymphocytic infiltration surrounding the necrosis, 3) periseptal or lobular panniculitis, and 4) microcalcifications. Other findings include changes of discoid lupus erythematosus in the overlying skin, lymphocytic vasculitis, mucin deposition, and hyalinization of the subepidermal papillary zones. The presence of these findings differentiates lupus panniculitis from other forms of panniculitis.[9] The classic finding in lupus mastitis is necrosis surrounding blood vessels, associated with heavy perivascular and periadenexal lymphocytic infiltrates and hyaline sclerosis of the dermal collagen. The presence of sclerosis and calcification are responsible for the hard, carcinoma-like feel of lupus mastitis and the unusual pattern of calcifications on mammography.

The differential diagnosis of lupus mastitis includes breast carcinoma, non-Hodgkin's lymphoma, uncommon manifestations of other connective tissue diseases (e.g., rheumatoid arthritis, polyarteritis nodosa, relapsing polychondritis, and Wegener's vasculitis), and idiopathic granulomatous mastitis. The clinical features and histology are helpful in differentiating between these conditions.[11]

Our case was straightforward, as she was a known case of SLE and the diffuse calcifications of a benign disease, suggestive of fat necrosis, were evident; however, the clinical presentation of constitutional symptoms, presence of submandibular swelling, axillary lymphadenopathy, hepatomegaly, and pericardial effusion made it necessary to rule out diseases like lymphoma, malignancy, and tuberculosis, and therefore we performed biopsy and mammography. Later, review of literature helped us to identify and understand not only the unique mammographic features but also the biopsy, FNA, CT scan, and USG findings.

Thus, in a clinical setting such as ours, the radiological and clinical features alone should suffice to make a diagnosis of lupus mastitis; FNA may help resolve doubts in unusual presentations and thus the complications of atrophic scarring and ulceration that are reported with biopsy can be avoided. With such an approach, it should be possible to avoid unnecessary surgeries and mastectomies and their consequences.[12]
